# Taxonomic composition and functional potentials of gastrointestinal microbiota in 12 wild-stranded cetaceans

**DOI:** 10.3389/fmicb.2024.1394745

**Published:** 2024-08-29

**Authors:** Jie Fan, Hui Kang, Meiqi Lv, Yuhuan Zhai, Yangyang Jia, Zixin Yang, Chengcheng Shi, Changhao Zhou, Lin Diao, Jingshuo Li, Xiaowei Jin, Shanshan Liu, Karsten Kristiansen, Peijun Zhang, Jianwei Chen, Songhai Li

**Affiliations:** ^1^College of Life Sciences, University of Chinese Academy of Sciences, Beijing, China; ^2^BGI Research, Qingdao, China; ^3^Marine Mammal and Marine Bioacoustics Laboratory, Institute of Deep-sea Science and Engineering, Chinese Academy of Sciences, Sanya, China; ^4^BGI Research, Shenzhen, China; ^5^China National Environmental Monitoring Centre, Beijing, China; ^6^Qingdao Key Laboratory of Marine Genomics, and Qingdao-Europe Advanced Institute for Life Sciences, BGI Research, Qingdao, China; ^7^Laboratory of Genomics and Molecular Biomedicine, Department of Biology, University of Copenhagen, Copenhagen, Denmark; ^8^The Innovation Research Center for Aquatic Mammals, and Key Laboratory of Aquatic Biodiversity and Conservation of the Chinese Academy of Sciences, Institute of Hydrobiology, Chinese Academy of Sciences, Wuhan, China

**Keywords:** stranded cetaceans, delphinids, physeteroids and ziphiid, gut microbiota, gastrointestinal microbiota, functional potentials, food digestion

## Abstract

Cetaceans play a crucial role in marine ecosystems; however, research on their gastrointestinal microbiota remains limited due to sampling constraints. In this study, we collected hindgut samples from 12 stranded cetaceans and performed 16S rRNA gene amplicon sequencing to investigate microbial composition and functional potentials. Analysis of ZOTUs profiles revealed that the phyla Firmicutes, Proteobacteria, and Bacteroidetes dominated all hindgut samples. However, unique microbial profiles were observed among different cetacean species, with significant separation of gut microbiota communities according to biological evolutionary lineages. Different genera that contain pathogens were observed distinguishing delphinids from physeteroids/ziphiids. Delphinid samples exhibited higher abundances of *Vibrio*, *Escherichia*, and *Paeniclostridium*, whereas physeteroid and ziphiid samples showed higher abundances of *Pseudomonas*, *Enterococcus*, and *Intestinimonas*. Functional analysis indicated convergence in the gut microbiota among all cetaceans, with shared bacterial infection pathways across hindgut samples. In addition, a comparison of the gastrointestinal microbial composition between a stranded short-finned pilot whale (*Globicephala macrorhynchus*) and a stranded rough-toothed dolphin (*Steno bredanensis*) using 16S rRNA gene sequencing revealed distinct microbial community structures and functional capacities. To the best of our knowledge, this study represents the first report on the gastrointestinal microbiota of the pantropical spotted dolphin (*Stenella attenuata*), Blainville’s beaked whale (*Mesoplodon densirostris*), and rough-toothed dolphin, with various comparisons conducted among different cetacean species. Our findings enhance the understanding of microbial composition and diversity in cetacean gastrointestinal microbiota, providing new insights into co-evolution and complex interactions between cetacean microbes and hosts.

## Introduction

Cetaceans, the largest known marine mammals, have evolved into approximately 90 species including whales, dolphins, and porpoises (Fordyce, 2018). As apex predators in the ocean, they play vital roles in marine ecosystems (Zhang and Goodman, 2023; Zhang et al., 2023a,b). Although the population of cetaceans has garnered global attention, their health status is facing multiple threats, including overfishing, habitat destruction, marine pollution, and pathogenic infections (Nachtsheim et al., 2021; Yuri and Findlay, 2021). These factors contribute to an uncertain outlook for these marine mammals. Recent studies even indicate that cetaceans under human care can live longer than their wild counterparts (Kirk-Cohen et al., 2023; Tidière et al., 2023). Previous research highlights the substantial influence of the mammalian gastrointestinal tract microbiota on various physiological processes including digestion, metabolism, tissue differentiation, pathogen resistance, and immunity (Sanders et al., 2015; Bik et al., 2016; Ost and Round, 2018; Yin et al., 2023). While extensive studies have explored the gastrointestinal microbiota of humans (Lin et al., 2023) and terrestrial mammals such as mice (Kieser et al., 2022), pigs (Hu et al., 2024), cattle (Xie et al., 2021), and giant pandas (Guo et al., 2020), research on cetacean gut microbiota remains limited (Sanders et al., 2015; Bik et al., 2016), particularly concerning microbial diversity and composition across different regions of their gastrointestinal tracts (Li et al., 2021).

Preliminary studies on the diversity, structure, and function of gut microbiota in cetaceans have revealed a highly diverse microbial community comprising more than 60 prokaryotic phyla with diverse functional profiles (Higgins, 2000; Waltzek et al., 2012). Over 400 prokaryotic species have been identified in the cetacean gut to date. The phyla Firmicutes, Proteobacteria, Bacteroidetes, and Fusobacteria are among the most abundant species in the cetacean gut microbiota, showing distinct differences from fish, terrestrial mammals, and seawater microbiota (Higgins, 2000; Waltzek et al., 2012). Significant variations in the diversity and composition of gut microbiota have been observed across different cetacean families. Specifically, the previous analysis revealed that toothed whales, alongside the North Pacific right whales (*Eubalaena japonica*), and different baleen whale species, exhibited markedly distinct abundances of *Mycobacterium*, *Cetobacterium*, *Bacillus*, and *Coprobacillus*, highlighting the diversity and specificity of microbial communities across these cetacean groups (Sanders et al., 2015). In addition, the gut microbiota of certain baleen whale species, such as the Pacific humpback whales (*Megaptera novaeangliae*) and bowhead whales (*Balaena mysticetus*), play critical roles in amino acid metabolism and synthesis, as well as lipid digestion (Sanders et al., 2015; Miller et al., 2020). Intestinal dysbiosis in cetaceans, triggered by factors, such as injury, exposure to organic pollutants, and bacterial infections, can result in host malnutrition, stranding events, and mortality (Nachtsheim et al., 2021; Yuri and Findlay, 2021). Pathogenic infections play a significant role in cetacean stranding, often indicating underlying health issues within these marine mammals (Waltzek et al., 2012; Di Guardo et al., 2018). Notable pathogens, such as *Brucella* (Guzmán-Verri et al., 2012)*, Aeromonas* (Pérez et al., 2015), and *Vibrio* (Buck and Spotte, 1986), are significant contributors to poor health in these animals, often associated with cetacean stranding. The cetacean gut microbiota is crucial for maintaining host health, particularly influencing disease susceptibility and immune system function (Ley et al., 2008; Krajmalnik-Brown et al., 2012; Hanning and Diaz-Sanchez, 2015; Lloyd-Price et al., 2019). However, current research on the cetacean gut microbiota remains constrained by small sample sizes of fewer than 10 individuals and limited species diversity. These limitations impede large-scale surveys and comprehensive comparative studies.

Compared to studies on the gut microbiota of cetaceans, research on the microbial composition of gastrointestinal tract tissues is even more infrequent due to the scarcity of anatomical samples from cetaceans. Only a few publications have documented the microbial composition of the stomach and foregut in cetaceans (Sanders et al., 2015; Monteiro et al., 2021; Wan et al., 2021, 2023). Preliminary analyses using amplicon sequencing and metagenomic approaches have revealed differences in microbial communities across different digestive tract regions of cetaceans, showing close functional interactions with their hosts (Erwin et al., 2017; Bai et al., 2022). For instance, studies have identified over 30 prokaryotic phyla in the gastrointestinal tracts of the common bottlenose dolphin (*Tursiops truncatus*), including two novel phyla, one named Delphibacteria, which suggests denitrification capabilities (Bik et al., 2016; Dudek et al., 2017). Moreover, Firmicutes predominated throughout the gastrointestinal tract of stranded pygmy sperm whales (*Kogia breviceps*) and dwarf sperm whales (*Kogia sima*), whereas Proteobacteria and Fusobacteria were abundant in the proximal gut, and Bacteroidetes emerged in the large intestine (Erwin et al., 2017; Li et al., 2021). Distinct core microbiota compositions have been reported for stomachs compared to the intestinal microbiota, featuring genera such as *Fusobacterium*, *Cetobacterium*, *Peptostreptococcus*, *Lactococcus*, and *Actinobacillus*, which are the dominant genera in the cetacean intestine (Miller et al., 2020).

In this study, we collected 19 gastrointestinal samples from 12 cetacean individuals representing 9 species. These included previously unreported species such as the pantropical spotted dolphin (*Stenella attenuata*), Blainville’s beaked whale (*Mesoplodon densirostris*), and rough-toothed dolphin (*Steno bredanensis*), alongside six species previously documented: Indo-Pacific humpback dolphin (*Sousa chinensis*), Risso’s dolphin (*Grampus griseus*), pygmy sperm whale (*Kogia breviceps*), dwarf sperm whale (*Kogia simus*), sperm whale (*Physeter macrocephalus*), and short-finned pilot whale (*Globicephala macrorhynchus*). We conducted 16S rRNA gene amplicon sequencing to explore microbial composition, diversity, and functional potential in the hindgut samples of cetaceans. Our investigation encompassed multiple perspectives, including host evolutionary relationships, stranding geographic locations, and different gastrointestinal tract tissues. Furthermore, we performed 16S and 18S rRNA gene amplicon sequencing on stomach, foregut, and hindgut samples from rough-toothed dolphins and short-finned pilot whales, providing new insights into the composition of prokaryotic and eukaryotic communities.

These data help address gaps in our understanding of the microbiota and its functions within the gastrointestinal tract of various cetacean species. They demonstrate how food sources may influence the gastrointestinal microbiota of cetaceans, supporting future research aimed at constructing a more comprehensive microbial overview for a larger number of cetacean species. Moreover, analysis of the gastrointestinal microbiota from multiple stranded cetaceans provides new insights into pathogenic detection and clinical health assessments of stranded individuals (Obusan et al., 2015).

## Materials and methods

### Sample collection

**Figure 1:**
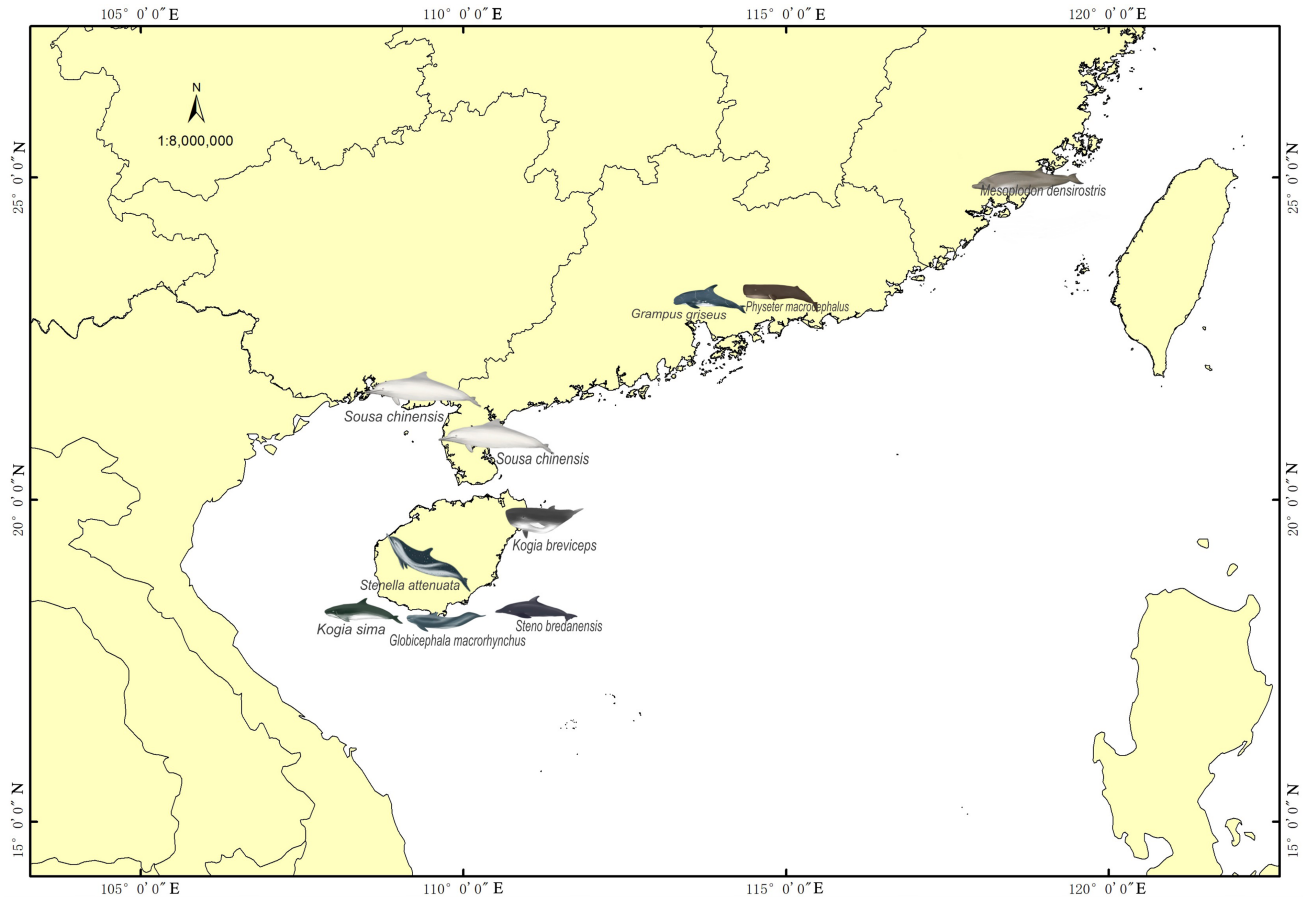
Stranded cetaceans sampling area. The red dots indicate the sampling locations in the coastal areas of southern China, mainly distributed in the Hainan Provinces, Guangdong Provinces, Fujian Provinces, and Guangxi Zhuang Autonomous Region.

Samples were collected from 12 stranded individuals of nine cetacean species in China between 2014 and 2019 (Figure 1 and Table 1). These individuals had been stranded in seven different cities. Four Indo-Pacific humpback dolphins, two from Qinzhou (Guangxi Zhuang Autonomous Region) and two from Zhanjiang (Guangdong Province), were found deceased and subsequently dissected for autopsies, from which hindgut samples were collected. The remaining eight cetaceans were initially alive when stranded in southern China but succumbed despite rescue efforts. The twelve stranded individuals were categorized into two groups based on their biological evolutionary lineages: the delphinid group, comprising eight Delphinidae individuals, and the physeteroid and ziphiid group, which included three Physeteroidea individuals and one Ziphiidae individual (Table 1). Standard post-mortem analyses were conducted on the deceased cetaceans, following established protocols. Hindgut samples were collected during necropsies for all individuals, while gastrointestinal samples, including foregut contents and stomach contents from a short-finned pilot whale and a rough-toothed dolphin, were also obtained and included in this study. All collected specimens were stored at −80°C until DNA extraction. Ethical approval for the collection of gastrointestinal samples in this study was granted by the Institute of Deep-sea Science and Engineering, Chinese Academy of Sciences, under ethical statement number SIDSSE-SYLL-MMMBL-01 and the Institutional Review Board of BGI (NO. BGI-R052-3 and NO. FT17160). All procedures adhered to ethical guidelines and legal requirements in China.

**Table 1:** Sampling information of 12 stranded cetaceans.

Normal name	Scientific name	Sample name	Taxonomy	Location	Death date	Sample date
Indo-Pacific humpback dolphin	*Sousa chinensis*	SCQZHG1	Delphinidae	Qinzhou, Guangxi	~2015.12.3	2015.12.5
	*Sousa chinensis*	SCQZHG2	Delphinidae	Qinzhou, Guangxi	~2015.12.3	2015.12.5
	*Sousa chinensis*	SCZJHG1	Delphinidae	Zhanjiang, Guangdong	~2017.11.8	2017.11.11
	*Sousa chinensis*	SCZJHG2	Delphinidae	Zhanjiang, Guangdong	~2017.11.8	2017.11.11
Pantropical spotted dolphin	*Stenella attenuata*	SAHG	Delphinidae	Ledong, Hainan	2016.8.16	A few days after frozen
Risso’s dolphin	*Grampus griseus*	GGHG	Delphinidae	Huizhou, Guangdong	2017.9.13	2017.9.13
Pygmy sperm whale	*Kogia breviceps*	KBHG	Physeteroidea	Wenchang, Hainan	2014.6.18	A few days after frozen
Dwarf sperm whale	*Kogia simus*	KSHG	Physeteroidea	Sanya, Hainan	2017.12.9	A few days after frozen
Sperm whale	*Physeter macrocephalus*	PMHG	Physeteroidea	Huizhou, Guangdong	2017.3.12	2017.3.13
Blainville’s beaked whale	*Mesoplodon densirostris*	MDHG	Ziphiidae	Xiamen, Fujian	2017.10.18	2017.10.18
Short-finned pilot whale	*Globicephala macrorhynchus*	GMHG, GMFG GMS1, GMS2	Delphinidae	Sanya, Hainan	2019.6.10	2019.6.11
Rough-toothed dolphin	*Steno bredanensis*	SBHG, SBFG SBS1, SBS2, SBS3	Delphinidae	Sanya, Hainan	2019.6.18	A few days after frozen

Postfix FG, HG, and S indicate foregut, hindgut, and stomach, respectively.

### DNA extraction and 16S rRNA gene amplicon sequencing of gastrointestinal samples

Approximately 10 g of gastrointestinal tract contents were collected from each cetacean individual. Each sample was homogenized and divided into three parts for DNA extraction. DNA extraction followed a modified CTAB protocol, and quantification was performed using a Qubit 3.0 Fluorometer. Due to the low microbial biomass in the intestinal contents of stranded cetaceans, each extraction did not meet the amplification requirements. Therefore, the three separate extractions were combined into one sample for subsequent PCR amplification. PCR amplification was carried out using the universal primers 515F-806R targeting the V4 region of the 16S rRNA gene (515F: GTGCCAGCMGCCGCGGTAA, 806R: GGACTACHVGGGTWTCTAAT). The PCR reactions were conducted using KAPA HiFi HotStart ReadyMix (2x) with primers and templates under the following conditions: initial denaturation at 95°C for 5 min, followed by 15 cycles of denaturation at 98°C for 20 s, annealing at 58°C for 30 s, extension at 72°C for 30 s, and a final extension at 72°C for 10 min (Jia et al., 2022). Subsequently, the purification of PCR products was performed using the GeneJET Gel Extraction Kit (Thermo Scientific). The PCR products with target bands were purified and pooled in equal masses to construct 16S amplicon libraries using the MGIEasy Universal DNA Library Prep kit v1.0 (PN: 1000006986), following the standard DNA nanoball (DNB) library construction protocol. All libraries were sequenced on the DNBSEQ-G400 platform with a paired-end sequencing mode with 200 bp read length at BGI Research (Qingdao, China) (Zhang Z. et al., 2023).

### Bioinformatic analysis of 16S amplicon data

The raw amplicon sequencing of paired-end reads was conducted using the EasyAmplicon pipeline (v1.0) (Liu Y. X. et al., 2023). Initially, FLASH (v1.2.11) (Magoč and Salzberg, 2011) merged the paired-end reads into tags with parameters “-min-overlap 10 -max-mismatch-density 0.1.” Primer sequences were then removed, and low-quality reads were filtered out using VSEARCH (v2.13.1) (Rognes et al., 2016) to generate clean tags. These clean tags were processed further to create zero-radius operational taxonomic units (ZOTUs) using the Unoise3 algorithm from USEARCH (Edgar and Flyvbjerg, 2015) for denoising (Liu G. et al., 2023). Taxonomic annotation of ZOTU representative sequences was performed using the sintax algorithm (Edgar, 2018) against the RDP training set (v18), employing a 0.8 confidence cutoff value. The abundance profile of ZOTUs was generated using the USEARCH “-otutab” command and normalized based on a standard sequence count derived from the sample with the average number of sequences, using the “-otutab_rare” command. Alpha diversity indices and beta diversity distances were computed using the vegan package (v2.6.4) within R software (v4.0.2). ZOTU sequences were aligned using PyNAST software, and a phylogenetic tree was constructed using FastTree (v2.1.5) (Price et al., 2010). Visualization of the phylogenetic tree was performed using iTOL (v5) (Letunic and Bork, 2019). The feature table used for linear discriminant analysis effect size (LEfSe) analysis was transformed by the EasyAmplicon pipeline. Subsequently, the LEfSe analysis was conducted and visualized by using the online tool ImageGP (Chen et al., 2022). The COI sequences of the nine cetaceans were downloaded from NCBI and then aligned by using MUSCLE (v3.8.31). The phylogenetic tree and distance matrix were measured by using Fasttree (v2.1.10). Finally, the Mantel test between the evolutionary distance matrix and microbial Bray Curtis distance matrix was conducted using the “mantel” function of the R package “vegan.”

### Functional predictions for prokaryotic communities

PICRUSt2 (Douglas et al., 2020) was employed to predict prokaryotic KEGG functions based on the taxonomy of 16S rRNA gene sequences, generating abundance profiles of KEGG Orthologs (KOs) and metabolic pathways. Statistical testing to discern significant differential KEGG pathways and enzymes among different cetaceans was performed using the edgeR package in R software (v4.0.2), and the results were visualized using OriginPro and ImageGP. Simultaneously, the Functional Annotation of PROkaryotic TAXa (FAPROTAX) database (Louca et al., 2016) was utilized to identify potentially pathogenic groups and detect experimentally verified pathogens from 16S rRNA gene sequences. Pathogens posing threats to human, aquatic animal, or plant welfare were categorized, including “animal parasites or symbionts,” “intracellular parasites,” “invertebrate parasites,” “human pathogens all,” “human pathogens diarrhea,” “human pathogens gastroenteritis,” “human pathogens meningitis,” “human pathogens nosocomial,” “human pathogens pneumonia,” “human pathogens septicemia,” “plant pathogen,” and “fish parasites.”

## Results

### Prokaryotic community overview of hindgut samples

**Figure 2:**
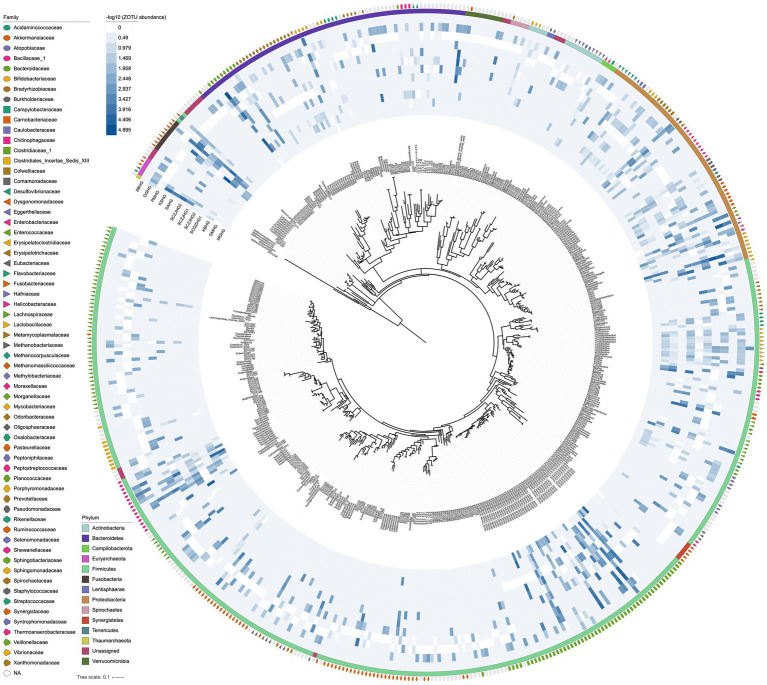
Phylogenetic tree of prokaryotic ZOTUs and distribution of relative abundances in the 12 hindgut samples. Relative abundance heatmap values are −log10 transformed. The outside circles represent the phylum and family taxonomic classification of the ZOTUs.

For the 12 hindgut samples collected from all stranded cetaceans, we obtained a total of 771,308 clean tags, with an average of 64,275 per sample. This analysis resulted in 535 prokaryotic ZOTUs, including 6 Archaea ZOTUs and 529 Bacteria ZOTUs (Figure 2, Supplementary Tables S1, S2). The rarefaction curves for each sample reached saturation, indicating that our sequencing strategy effectively captured the microbial taxa in all samples (Supplementary Figure S1). We identified 19 core ZOTUs, primarily from Firmicutes (10 ZOTUs) and Proteobacteria (6 ZOTUs), present in ≥75% of the hindgut samples (Supplementary Table S1). In addition, 184 specific ZOTUs, constituting approximately 34.39% of all ZOTUs, appeared in only one of the twelve samples, with the majority (116/535, 21.68%) being unclassified Firmicutes (Supplementary Table S1). The 19 core ZOTUs were also the most abundant across all hindgut samples, with an average relative abundance of 23.81% for each ZOTU, whereas the 183 specific ZOTUs were rare, exhibiting extremely low abundances (average ~ 0.01%). This indicates a significant overlap in the core gut microbiota among different cetaceans.

The gut microbial compositions of the three cetacean species, sequenced for the first time, varied significantly (Figure 2). In the Blainville’s beaked whale, the dominant genus was *Bacteroides* (76.8%), followed by *Clostridium sensu stricto* (7.43%), *Vibrio* (5.13%), *Parabacteroides* (4.58%), *Morganella* (2.46%), and *Providencia* (2.11%). In the pantropical spotted dolphin, the most abundant genera were *Peptostreptococcus* (41.2%), *Clostridium sensu stricto* (20.1%), *Kurthia* (12.7%), *Enterococcus* (4.74%), and *Actinobacillus* (2.64%), along with a substantial fraction of unclassified microbes (16.3%). The rough-toothed dolphin exhibited a distinct microbial profile compared to the pantropical spotted dolphin, with the top five genera being *Vibrio* (39.5%), *Bacteroides* (28.1%), *Cetobacterium* (13.9%), *Clostridium sensu stricto* (7.47%), and *Paeniclostridium* (2.70%).

### Gut microbiota differences between the delphinids, and physeteroids, and ziphiid

**Figure 3:**
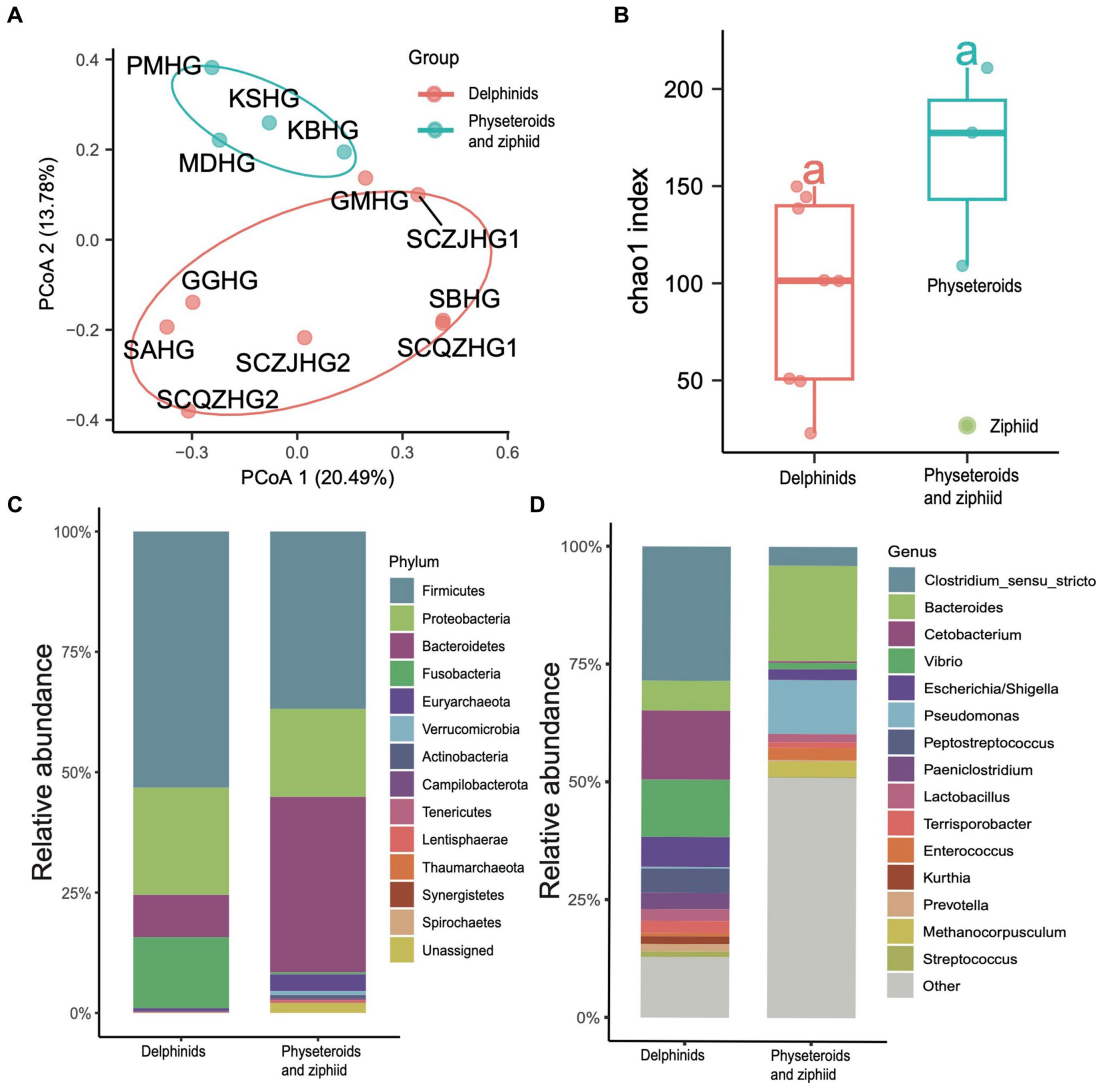
Gut microbiota composition differs among cetacean individuals. **(A)** PCoA of 12 hindgut samples at the ZOTU level based on Bray Curtis distances. **(B)** Boxplot showing the comparison of the α-diversity indices Chao1 index between the eight delphinid samples and four physeteroid and ziphiid samples. **(C,D)** The stacked bar chart shows the average prokaryotic relative abundance of hindgut samples at the phylum and genus level of the two cetacean lineages. **(C)** A comparison of the microbiota of delphinids, physeteroids, and ziphid and the phylum level. A total of 13 phyla were detected, while the unclassified taxa were named “Unassigned.” **(D)** A comparison of the microbiota of delphinids, physeteroids, and ziphid and the genus level. A total of 128 genera were detected, only the top 15 most abundant genera are shown here, and the remaining genera including unclassified taxa were grouped as “Other.”

We conducted principal coordinate analysis (PCoA) based on the Bray Curtis β-diversity indices for the hindgut samples. The PCoA results revealed a separation in microbial profiles aligned with the evolutionary relationships of the cetacean species. Specifically, the hindgut microbiota communities of delphinid samples (Delphinidae cetaceans) significantly differed from those of physeteroid and ziphiid samples (Physeteroidea and Ziphiidae cetaceans) (ANOSIM test: 999 permutations, *p* < 0.05) (Figure 3A). To reveal the relationship between the distance between hindgut microbial samples and the evolutionary distance of the cetaceans, we constructed the COI phylogenetic tree of the nine cetacean species, and then, we observed two separated clades between the delphinids and the physeteroids and ziphiid (Supplementary Figure S2). Mantel test analysis between the evolutionary distance matrix and hindgut microbial Bray Curtis distance matrix revealed phylosymbiosis signatures of the hindgut microbial composition across different cetacean species (R = 0.51, *p* = 0.04). The gut microbiota of the ziphiid Blainville’s beaked whale (MDHG) was more similar to that of physeteroids, differing from delphinids. Conversely, the gut microbial compositions of the pantropical spotted dolphin (SAHG) and rough-toothed dolphin (SBHG) were similar to those of other delphinids. We also calculated the α-diversity indices to evaluate microbial community diversity in the hindgut samples, finding that the Chao1 index was notably higher in physeteroid samples (average = 165.80) compared to delphinid samples (average = 94.88), although this difference was not statistically significant (ANOVA test, adjusted *p* > 0.05) (Figure 3B).

The major phyla identified in the three physeteroid hindgut samples and one ziphiid sample were Firmicutes (36.70%), Bacteroidetes (36.58%), Proteobacteria (17.81%), and Euryarchaeota (3.42%). In contrast, the eight delphinid samples were predominantly composed of Firmicutes (53.04%), Proteobacteria (20.84%), Fusobacteria (14.67%), and Bacteroidetes (8.72%) (Figure 3C). Physeteroid and ziphiid samples exhibited a higher abundance of Bacteroidetes, whereas the delphinid samples demonstrated a greater prevalence of Firmicutes and Fusobacteria. Despite differences in microbial distributions at the phylum level, both groups showed a relatively high proportion of Proteobacteria. At the genus level, the dominant genera in all cetacean hindgut samples were *Clostridium sensu stricto* (20.40%), *Bacteroides* (10.96%), and *Cetobacterium* (9.83%) (Figure 3D). However, the genera *Vibrio*, *Escherichia*, and *Paeniclostridium* were more abundant in delphinids, with average relative abundances of 12.28, 6.25, and 3.44%, respectively, compared to 1.43, 2.20, and 0.04% in the physeteroid and ziphiid samples. In contrast, the physeteroid and ziphiid samples had a higher abundance of *Pseudomonas* (11.15%), *Enterococcus* (1.14%), and *Intestinimonas* (0.74%) than the delphinids, which had average relative abundances of 0.32, 0.59, and 0.88%, respectively. In addition, unclassified prokaryotic communities accounted for an average of 12.28% of the total relative abundance at the genus level, indicating that much remains unknown about the cetacean gut microbiota.

### Microbial functional convergence in cetacean hindgut samples

**Figure 4:**
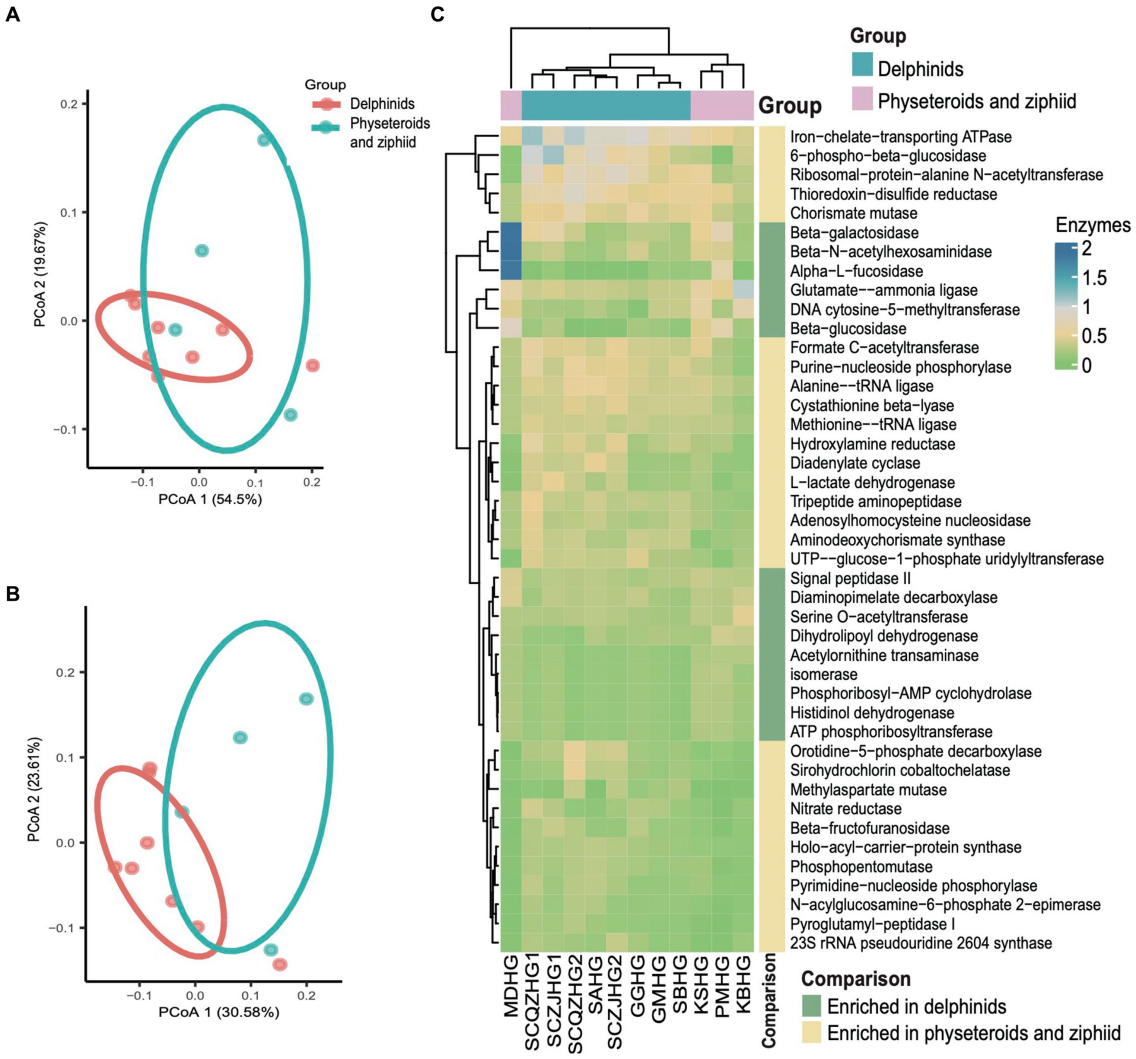
Predicted functional potential of the cetacean hindgut microbiota. **(A,B)** PCoA based on the Bray Curtis distance of KEGG pathways and enzyme functional abundance profiles, respectively. **(C)** A total of 43 enzymes were over-represented for the two cetacean lineages.

Kyoto Encyclopedia of Genes and Genomes (KEGG) pathway enrichment analysis was performed on the 12 hindgut samples to generate the KEGG functional abundance profile. The results revealed a total of 162 pathways across all cetacean samples. Notably, pathways related to the biosynthesis of ansamycins and fatty acid biosynthesis were abundant in all hindgut samples. PCoA indicated that the KEGG pathway abundances in cetacean microbiota were convergent between the delphinid and the physeteroid/ziphiid samples (PERMANOVA test: 999 permutations, *p* > 0.05) (Figure 4A). Only nine of the 162 pathways showed significant differences in abundance between the two cetacean lineages. Specifically, pathways, such as glycosaminoglycan degradation, glycine, serine, threonine, and histidine metabolism and sphingolipid metabolism, were significantly enriched in the physeteroid and ziphiid samples, while pathways, such as glycerophospholipid metabolism, phosphotransferase system (PTS), and D-alanine metabolism, were significantly enriched in the delphinids (*p* < 0.05) (Supplementary Table S3). Furthermore, the abundance profile of enzymes revealed significant variation between samples (PERMANOVA test: 999 permutations, *p* < 0.05) (Figure 4B). We identified a total of 15 enzymes that were more abundant in the delphinid samples, including ATP phosphoribosyltransferase and beta-N-acetylhexosaminidase, which are related to energy and carbohydrate metabolism. Conversely, 28 enzymes, such as diadenylate cyclase and ribosomal-protein-alanine N-acetyltransferase, which are related to nucleic acid and protein metabolism, were more abundant in the physeteroid and ziphiid samples (*p* < 0.05) (Figure 4C).

Moreover, six KEGG pathways, including those related to *Vibrio cholerae* infection and the *Vibrio cholerae* pathogenic cycle, were found to have a combined total abundance of 7.04% across all hindgut samples (Supplementary Figure S3). In addition, we identified 24 ZOTUs with a relative abundance of approximately 12.21% that were assigned to potential pathogenic groups classified as “animal parasites or symbionts” and “human pathogens all” according to the FAPROTAX database. These potentially pathogenic ZOTUs included species from *Prevotella* (14 ZOTUs), *Actinobacillus* (4 ZOTUs), other *Pasteurellaceae* (3 ZOTUs), *Stenotrophomonas* (2 ZOTUs), *Escherichia* (2 ZOTUs), and *Roseburia* (1 ZOTU) (Supplementary Table S4). The potential pathogenic *Prevotella* and *Stenotrophomonas* were widespread across all hindgut samples of sampled cetaceans, with a notably higher relative abundance in sample ZJBHT1, which is a sample of *Sousa chinensis*.

### *Sousa chinensis* stranded at two locations harbor distinct microbial communities

**Figure 5:**
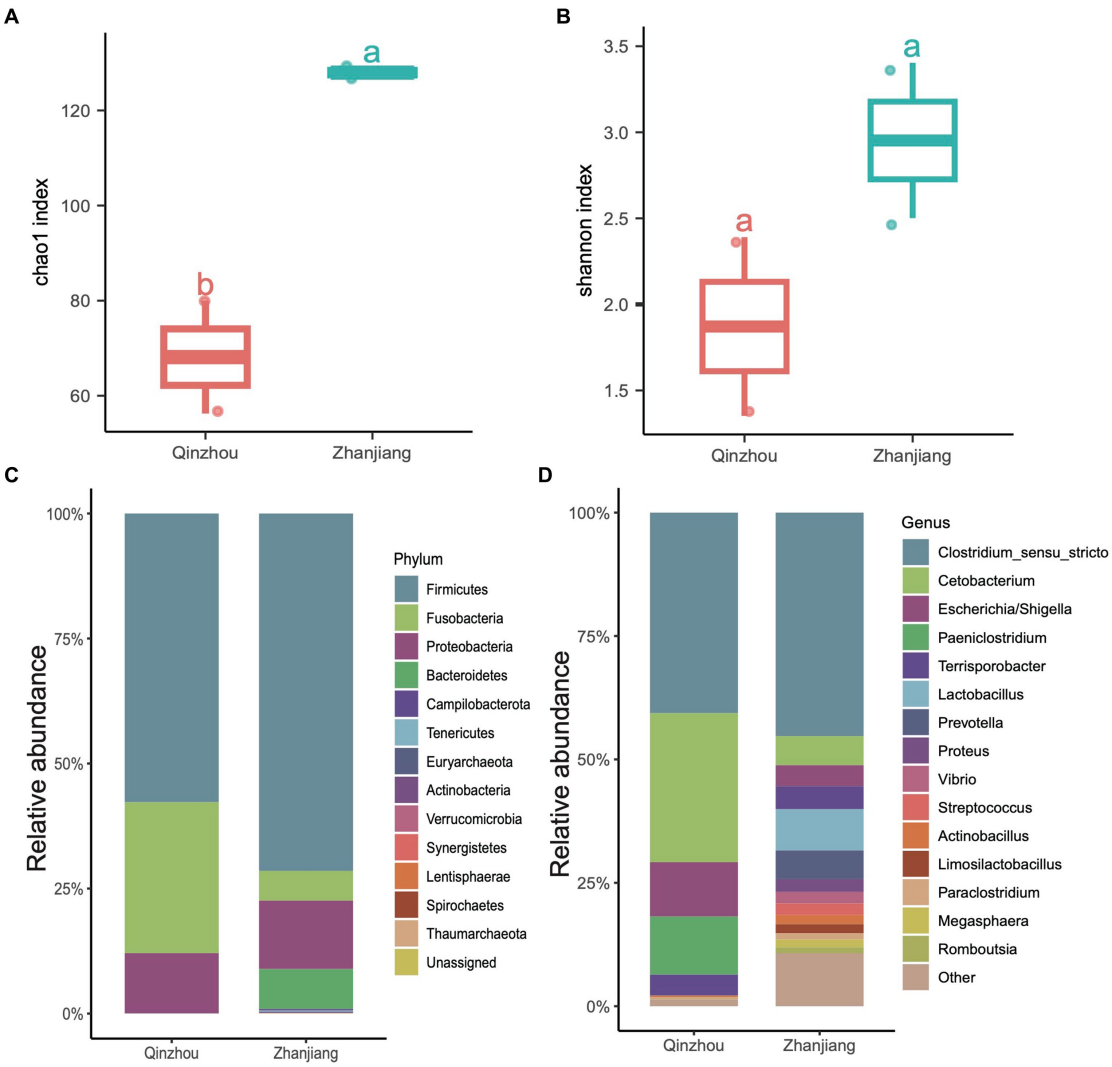
Taxonomic composition of four Indo-Pacific humpback dolphins. **(A,B)** Comparisons of α-diversity indices, including Chao1 index **(A)** and Shannon index **(B)**, of the four Indo-Pacific humpback dolphins stranded in Qinzhou City and Zhanjiang City. **(C,D)** Stacked bar chart showing the average relative abundance at the phylum **(C)** and genus **(D)** levels for the four Indo-Pacific humpback dolphin hindgut samples.

We further analyzed the hindgut microbial composition of the four Indo-Pacific humpback dolphins (*Sousa chinensis*) stranded in Zhanjiang City (Leizhou Bay colony) and Qinzhou City (Sanniang Bay colony). We observed significant differences in the Chao1 indices between the two locations (ANOVA test, adjusted *p* < 0.05) (Figure 5A), and although the Shannon indices of Indo-Pacific humpback dolphins stranded in Zhanjiang were higher than those in Qinzhou, the difference was not statistically significant (Figure 5B). We also calculated the relative abundances at the phylum and genus levels to provide detailed information on the microbial community composition. At the phylum level, samples from Zhanjiang City were predominantly composed of Firmicutes (~72.20%) with a notable presence of Bacteroidetes (~6.76%), while samples from Qinzhou City were dominated by Firmicutes (~56.55%) and Fusobacteria (~30.25%) with remarkably fewer Bacteroidetes (~0.01%) (Figure 5C). The common abundant genus across all four samples was *Clostridium sensu stricto* (average ~ 42.60%). The genera *Cetobacterium* (30.25% vs. 5.8%), *Escherichia* (12.10% vs. 4.52%), and *Paeniclostridium* (11.54% vs. 0.18%) were more dominant in samples from Qinzhou City, whereas *Terrisporobacter* (3.90% vs. 5.16%) and *Lactobacillus* (0.02% vs. 8.34%) were more abundant in samples from Zhanjiang City (Figure 5D). Regarding α-diversity analysis (richness and Shannon indices), both indices were notably higher in the gut microbiota of dolphins from Zhanjiang City compared to those from Qinzhou City. We also found several genera that were abundant in Zhanjiang dolphins, such as *Prevotella*, *Actinobacillus*, *Streptococcus*, *Proteus*, and *Vibrio*, each with a relative abundance of over 2%, but these were rarely or not observed in Qinzhou dolphins (Figure 5D).

### Microbial community differentiation in the gastrointestinal tract of two different delphinids

**Figure 6:**
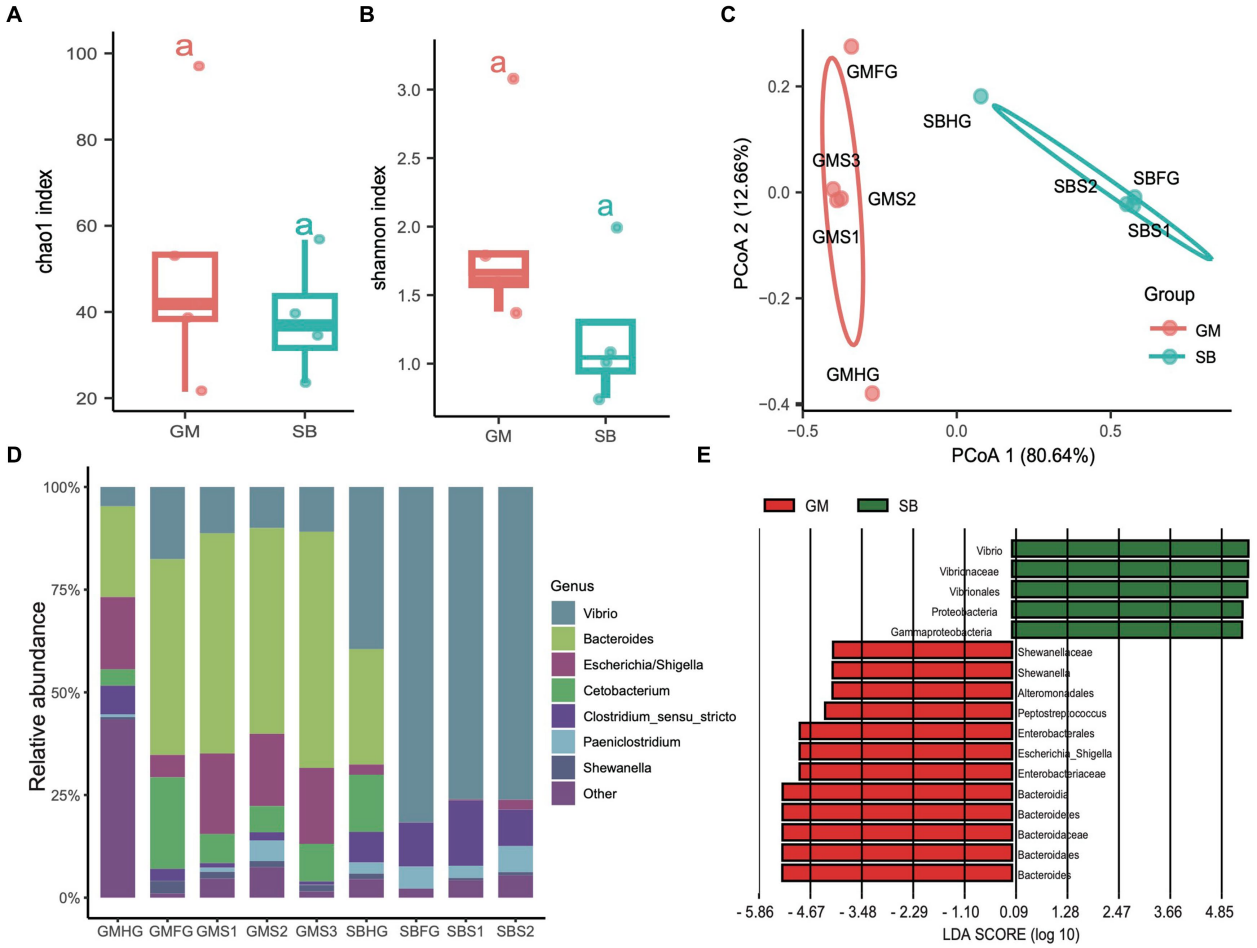
Comparisons of the prokaryotic diversity and composition in the gastrointestinal tract of rough-toothed dolphin and short-finned pilot whales. **(A,B)** Boxplots showing the α-diversity indices comparisons, including Chao1 index **(A)** and Shannon index **(B)**, of the gastrointestinal tract samples from rough-toothed dolphin (SB) and four from the short-finned pilot whale (GM). **(C)** PCoA based on the Bray Curtis distance to visualize the structure of prokaryotic communities in the stomach (GMS1, GMS2, and GMS3), foregut (GMFG), and hindgut (GMHG) of GM samples and stomach (SBS1 and SBS2), foregut (SBFG), and hindgut (SBHG) of SB samples. **(D)** Stacked bar chart showing the prokaryotic relative abundance in the stomach, foregut, and hindgut samples of GM and SB at the genus level. **(E)** Prokaryotic biomarkers significantly enriched in the GM and SB gastrointestinal tract samples determined by the linear discriminant analysis effect size (LEfSe) analysis.

In addition to examining the differences in the microbial communities of hindgut samples among different cetaceans, we investigated the community composition and structure of the stomach and foregut microbiota in two delphinid species: the rough-toothed dolphin (*Steno bredanensis*, SB) and the short-finned pilot whale (*Globicephala macrorhynchus*, GM). After quality assessment, we obtained a total of 443,154 clean tags from five gastrointestinal tract samples from rough-toothed dolphins and four from short-finned pilot whales, resulting in the identification of 139 ZOTUs (Supplementary Tables S1, S2). We calculated the α-diversities of microbial communities in the gastrointestinal tracts of these two Delphinidae species. The Chao1 and Shannon indices indicated that the α-diversity of the gastrointestinal microbiota in GM was consistently higher than in SB, although this difference was not statistically significant (ANOVA test, adjusted *p* > 0.05) (Figures 6A,B). However, PCoA based on Bray Curtis distances revealed distinct separation between the two species, suggesting they harbor different gastrointestinal microbial communities (PERMANOVA test: 999 permutations, SumsOfSqs = 7.79, *F* = 21.50, *p* < 0.05) (Figure 6C).

The genera *Bacteroides* (range 28.20 to 55.00%), *Vibrio* (10.10 to 39.60%), and *Escherichia* (2.40 to 19.90%) were predominant in the GM samples, whereas the SB samples were dominated by the genera *Vibrio* (4.69 to 81.40%), *Clostridium sensu stricto* (6.28 to 16.20%), and *Paeniclostridium* (0.81 to 6.03%) (Figure 6D and Supplementary Table S1). We also conducted LEfSe analysis to identify significant biomarkers distinguishing the two delphinid species. The result revealed that genera or families related to *Vibrio* and *Escherichia*, which are known to potentially cause infections and diseases (Di Renzo et al., 2017), were particularly abundant in SB and GM, respectively (Figure 6E).

**Figure 7:**
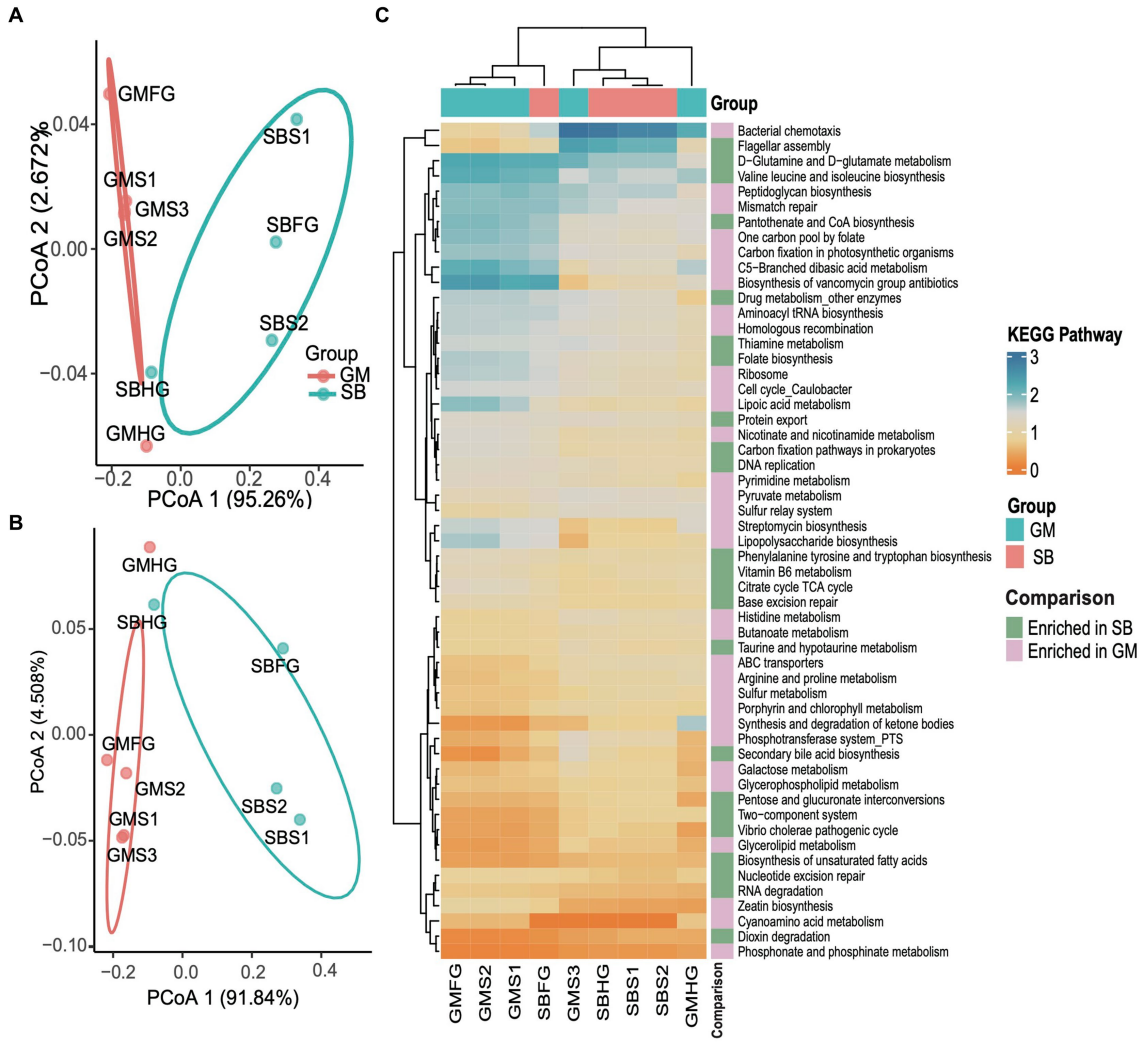
Comparative analyses of potential functions in the stomach, foregut, and hindgut of the short-finned pilot whale (GM) and rough-toothed dolphin (SB) gastrointestinal samples. **(A)** PCoA based on the Bray Curtis distance of the KEGG pathway functional profiles of the two delphinids. **(B)** PCoA based on the Bray Curtis distance of the enzyme profiles. **(C)** Relative abundance heatmap of 55 KEGG pathways significantly enriched in stomach samples from the two delphinids.

KEGG pathways predicted by PICRUSt2 revealed a total of 141 pathways across the nine gastrointestinal samples. Functional profiling indicated that the hindgut samples from the two delphinid species exhibited similar compositions, as did the foregut samples. However, we observed significant differences in the functional composition of the gastrointestinal samples between the two delphinid species based on the KEGG pathway abundance profile (PERMANOVA test: 999 permutations, SumsOfSqs = 0.028, *F* = 11.54, *p* < 0.05) and the enzyme abundance profile (PERMANOVA test: 999 permutations, SumsOfSqs = 0.089, *F* = 11.37, *p* < 0.05), and the stomach samples between the two species were significantly separated (Figures 7A,B). Using edgeR, we identified significant pathways that differed significantly in abundance between stomach samples from the two species (*p* < 0.05). The analysis revealed that 32 pathways, including glycerolipid metabolism and glycerophospholipid metabolism, were enriched in the GM stomach samples. In contrast, 23 pathways, such as the *Vibrio cholerae* pathogenic cycle and carbon fixation pathways in prokaryotes, were enriched in the SB stomach samples (Figure 7C).

## Discussion

Cetaceans, as top predators, play a crucial role in marine ecosystems, and their distribution can indicate prey availability and overall ocean health. However, many cetacean species face threats and are highly sensitive to anthropogenic influences such as bycatch in fishing gear, ship strikes, hunting, chemicals, noise pollution, and environmental changes across their habitats (Coombs et al., 2019). The composition and structure of their gastrointestinal microbiota are often species- and tissue-specific and are closely related to the physiological conditions. The microbiota can vary significantly in response to the health status of the host, and even minor disruptions can lead to microbial dysfunction and potentially result in host illness (Li C. et al., 2022). To better characterize the gastrointestinal microbiota of different cetaceans, we conducted 16S rDNA amplicon sequencing on 19 gastrointestinal samples from 12 stranded cetaceans. Consistent with previous studies (Li et al., 2019, 2021; Wan et al., 2021), Firmicutes, Bacteroidetes, Proteobacteria, and Fusobacteria were the dominant phyla in cetacean intestines (Figure 3C). The genera *Cetobacterium*, *Lactobacillus*, *Pseudomonas*, and *Clostridium sensu stricto* were the most prevalent commensal bacteria across all hindgut samples (Figure 3D). These results provide insights into the gastrointestinal microbiota of cetaceans, highlighting the dominant bacterial phyla and prevalent symbiotic genera. Understanding these microbial communities can help assess cetacean health and the impact of environmental changes on marine ecosystems.

In addition to normal commensals, we identified a large number of potentially pathogenic bacteria, including *Paeniclostridium, Escherichia,* and Pasteeurellaceae, as well as pyogenic bacteria such as *Pseudomonas*, *Vibrio*, and *Streptococcus*, which are commonly associated with inflammation. These genera comprise several known potential pathogens or pro-inflammatory bacteria. For example, *Paeniclostridium sordellii*, *Pseudomonas aeruginosa*, *Vibrio cholerae*, and *Vibrio vulnificus*, which are widely distributed in soil, water, plants, and animals, are bacterial pathogens that may cause serious, even lethal infections in humans (Lin et al., 2023). In addition, the family Pasteeurellaceae and the genus *Streptococcus* comprise some opportunistic pathogens, such as the species *Proteus mirabilis*, *Proteus vulgaris*, and *Proteus penneri*, from Pasteeurellaceae, and the species *S. pyogenes*, *S. faecalis*, and *S. suis* from *Streptococcus*. These species are occasionally found in the gastrointestinal tract of humans, pigs, and different mammalian species causing diarrhea or inflammation (Lin et al., 2023; Hu et al., 2024). Notably, a previous study indicated that Pasteurellaceae and *Paeniclostridium* were abundant in the intestines of dwarf sperm whales, potentially acting as opportunistic pathogens causing acute infections (Li X. et al., 2022). Furthermore, *Pseudomonas* and *Vibrio* were possibly contributing to infections that may lead to stranding or death (Waltzek et al., 2012; Di Guardo et al., 2018). The presence of both pathogenic and pyogenic bacteria has been linked to the compromised health of cetaceans, and their dominance in the samples suggests a possible link to the unhealthy condition of the stranded cetaceans (Waltzek et al., 2012; Di Guardo et al., 2018). A comprehensive understanding of pathogenic bacteria in cetacean intestinal microbiota is pivotal for effective disease prevention and treatment strategies, thereby forming a critical foundation for cetacean conservation efforts.

Functional predictions indicated that even though different cetacean lineages have different hindgut microbiota composition (Figures 2, 3A), their functional potential was similar without significant separation between different cetacean lineages, indicating that hindgut bacterial functions in cetaceans exhibit marked similarities (Figures 4A,B). The KEGG pathways in hindgut samples from delphinids, physeteroids, and ziphiids showed convergence, with only a few pathways differing between the groups. Similar results were observed in four-stranded Indo-Pacific humpback dolphins from two different locations (Qinzhou vs. Zhanjiang) (Figure 5). Lipids are crucial diagnostic indicators of the metabolic and physiological status of cetaceans, and lipid-related metabolic functions observed consistently in all hindgut samples may reflect the digestion of lipid-rich preys and the absorption of wax (Tang et al., 2018; Miller et al., 2020; Monteiro et al., 2021). Physeteroid and ziphiid hindgut samples exhibited higher abundances of pathways related to glycosaminoglycan degradation and histidine metabolism, whereas glycerophospholipid metabolism was significantly enriched in delphinid samples (Figure 4C). Our results suggest that not only terrestrial mammals but also marine mammals have developed persistent and specific interactions with the hindgut microbiota during evolution. We also investigated the spatial microbial composition and function along the gastrointestinal tracts of a rough-toothed dolphin (SB) and a short-finned pilot whale (GM). Although significant differences in the gastrointestinal microbiota were observed between individuals (Figures 6D,E), the functional analysis indicated that while there were distinct functional profiles in the stomach and foregut samples, the hindgut samples exhibited similar functions (Figures 7A,B). The different functional potential of the stomach microbiota possibly reflects the ingestion of different food resources. In addition, except for different food sources, these differences may also be attributable to the distinct physiological roles of the digestive tract in the two species (Krajmalnik-Brown et al., 2012).

Due to challenges in studying the natural feeding habits of cetaceans, metabarcoding sequencing of the gastrointestinal tracts from stranded cetaceans has become a primary method for exploring their diets (Zhang X. et al., 2023). Previous studies have shown that pilot whales typically consume at least 18 species of cephalopods (including those from the families Octopodidae and Ommastrephidae), as well as fish, crustaceans, and other mollusks. In contrast, rough-toothed dolphins tend to feed on cephalopods and large fish such as saury, needlefish, and mahimahi (Barros et al., 2004; Santos et al., 2014; Beasley et al., 2019). We performed 18S rDNA and 12S rDNA sequencing on the gastrointestinal tract samples from the GM and SB to analyze the composition of eukaryotic taxa as previously described (Supplementary material S1) (Zou et al., 2020; Chen et al., 2021, 2024). Our results indicated that Ochrophyta was relatively abundant in both the stomach and gut samples of these cetaceans, along with various candidate food species, including marine fish, mollusks, and cephalopods (Supplementary Figure S4 and Supplementary Table S5). Most of the detected species appeared to be from natural sources rather than artificial foods, indicating that they are likely part of the natural diet of these delphinid species. However, further comparative tests are necessary to substantiate this notion. The differences in gastrointestinal eukaryotes likely reflect the host’s diet and may also influence their microbial communities.

The composition and function of gastrointestinal microbiota in cetaceans play a crucial role in maintaining host health, particularly in relation to disease manifestation and the proper functioning of the immune system. An imbalance in the intestinal micro-ecology and the production of harmful microbial metabolites can lead to host inflammation, impaired immune responses, malnutrition, and various diseases (Li et al., 2019; Nachtsheim et al., 2021). The microorganisms in the cetacean digestive tract may not only provide valuable insights into host health but also reflect immune status, evolutionary history, habitat, and dietary habits, all of which can influence the composition and distribution of the gastrointestinal microbiota (Bik et al., 2016; Bai et al., 2021; Levin et al., 2021). Our study offers insights into the composition and function of the gastrointestinal microbiota in cetaceans, addressing significant gaps in our knowledge of the gut microbiota across various whale species. However, the current understanding of this field remains limited. To deepen our comprehension of the structure and assembly mechanisms of cetacean gastrointestinal microbial communities, future research should focus on examining the spatiotemporal distribution of these microbiota across multiple individuals of the same cetacean species, as well as among different cetacean species.

Since our study was based on stranded cetaceans (12 individuals) and relied on gut microbiota samples that may not represent healthy, free-ranging populations, our study has some limitations. Some data were collected shortly after attempted rescue and exposure to artificial diets and antibiotics, which could alter the microbiota and may not reflect natural conditions. The SB and GM individuals were subjected to attempted rescue and were given artificial foods and antibiotics for approximately 10 days before samples were collected immediately after their deaths. The reliance on 16S rDNA sequencing constrains our ability to explore functional genomics and other microbial communities such as viruses and fungi. The absence of long-term, spatiotemporal sampling restricts understanding of microbiota dynamics over time and across different habitats. Future research should include larger, diverse sample sizes, incorporate healthy control groups, and extend to longitudinal studies.

## Data Availability

The data that support the findings of this study have been deposited into the China National GeneBank Sequence Archive (CNSA) of China National GeneBank DataBase (CNGBdb) with accession numbers CNP0005144.
