# Correction: Taxonomic composition and functional potentials of gastrointestinal microbiota in 12 wild-stranded cetaceans

**DOI:** 10.3389/fmicb.2025.1754929

**Published:** 2025-12-12

**Authors:** Jie Fan, Hui Kang, Meiqi Lv, Yuhuan Zhai, Yangyang Jia, Zixin Yang, Chengcheng Shi, Changhao Zhou, Lin Diao, Jingshuo Li, Xiaowei Jin, Shanshan Liu, Karsten Kristiansen, Peijun Zhang, Jianwei Chen, Songhai Li

**Affiliations:** 1 College of Life Sciences, University of Chinese Academy of Sciences, Beijing, China; 2 BGI Research, Qingdao, China; 3 Marine Mammal and Marine Bioacoustics Laboratory, Institute of Deep-sea Science and Engineering, Chinese Academy of Sciences, Sanya, China; 4 BGI Research, Shenzhen, China; 5 China National Environmental Monitoring Centre, Beijing, China; 6 Qingdao Key Laboratory of Marine Genomics, and Qingdao-Europe Advanced Institute for Life Sciences, BGI Research, Qingdao, China; 7 Laboratory of Genomics and Molecular Biomedicine, Department of Biology, University of Copenhagen, Copenhagen, Denmark; 8 The Innovation Research Center for Aquatic Mammals, and Key Laboratory of Aquatic Biodiversity and Conservation of the Chinese Academy of Sciences, Institute of Hydrobiology, Chinese Academy of Sciences, Wuhan, China

**Keywords:** stranded cetaceans, delphinids, physeteroids and ziphiid, gut microbiota, gastrointestinal microbiota, functional potentials, food digestion

The author list contained a misspelling of Jingshuo Li's name, which was written as “Jingsuo Li”.

The original version of this article has been updated.

