# Supplementary material for: Taxonomic composition and functional potentials of gastrointestinal microbiota in 12 wild-stranded cetaceans
Source: Front Microbiol. 2024 Aug 29;15:1394745. doi: 10.3389/fmicb.2024.1394745 (PMC11390675; doi:10.3389/fmicb.2024.1394745)
Supplement: Supplementary file 2 [file Data_Sheet_1.pdf]

Supplementary Information

Supplementary Note 1

Eukaryotic sequencing and data analysis of two dolphin species

Amplification of the 18S rDNA V4 region was conducted by using the eukaryotic primers 528F-706R (528F: GCGGTAATTCCAGCTCCAA, 706R: AATCCRAGAATTTCACCTCT) for the four gastrointestinal samples from rough-toothed dolphin and five samples from the short-finned pilot whale (**Table 1**). The amplification, sequencing strategy and sequencing data processes were the same as for the 16S rDNA gene amplicon sequencing described previously. Furthermore, the clean tags were clustered into operational taxonomic units (OTUs) using the USEARCH "-cluster_otus" command and then generated the OTU abundance profile using the "--usearch_global" command with sequence identity ≥ 0.97. After removing the chimeric OTUs, the OTU representative sequences were taxonomically classified using the syntax algorithm against the PR2 database (v4.12.0) with a confidence threshold of 0.8. Besides, the mitochondrial 12S rDNA primers MiFish-F (GTCGGTAAAACTCGTGCCAGC) and MiFish-R (CATAGTGGGGTATCTAATCCCAGTTTG) were performed to identify fish species that occurred in the gastrointestinal tract samples as previously described. Briefly, the 12S rDNA amplicon libraries were sequenced on the BGISEQ-500 platform with a 150 bp paired-end model. The clean tags were clustered to OTUs with a 97% threshold, and then taxonomically annotated against the non-redundant nucleotide (nt) database and the MitoFish database using BLAST+ (v2.2.6).

Supplementary Figures

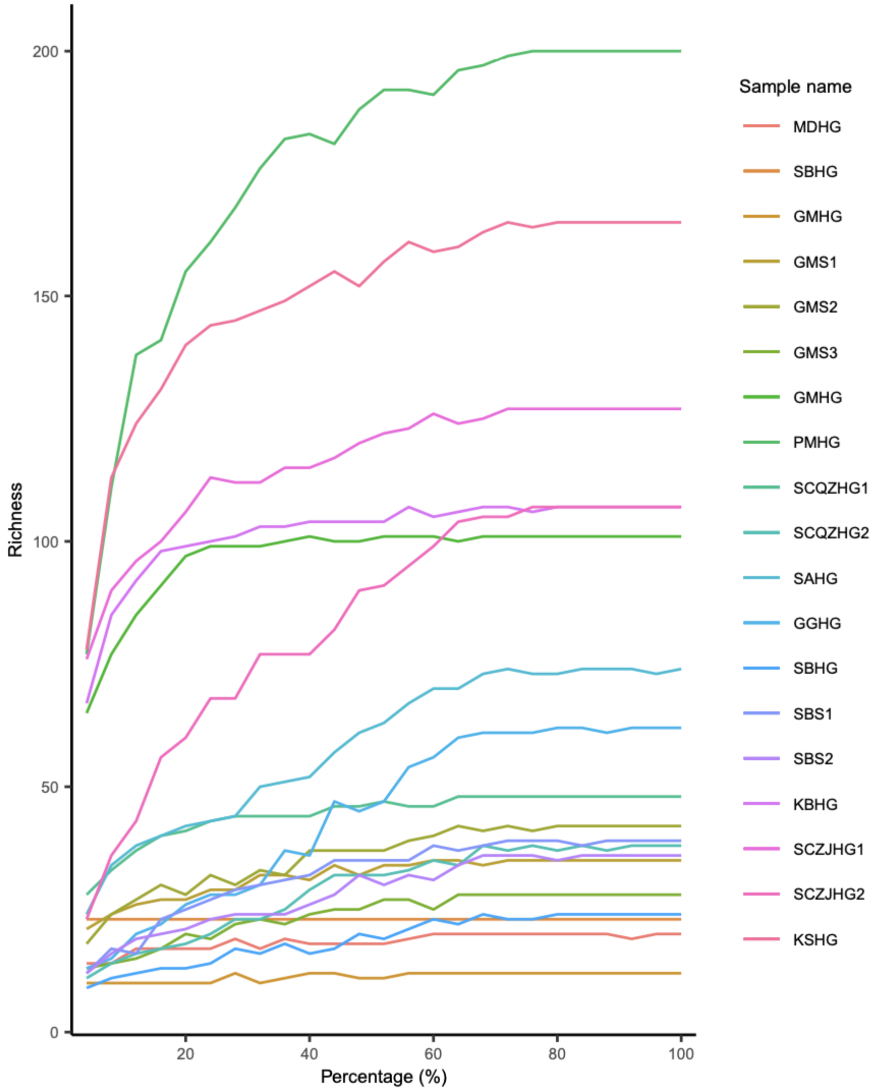

Figure S1. The rarefaction curve of all sample sequences.

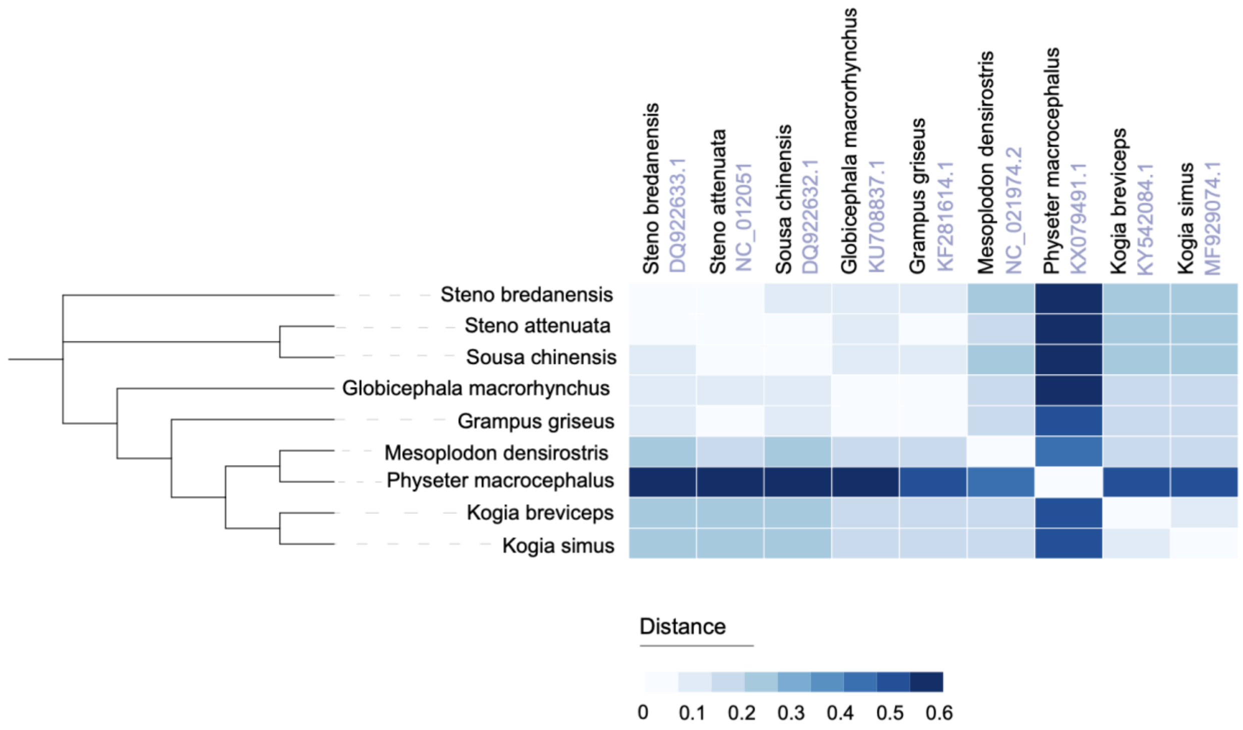

Figure S2. The phylogenetic tree (left) and evolutionary distance heatmap (right) of nine cetacean species based on the COI genes.

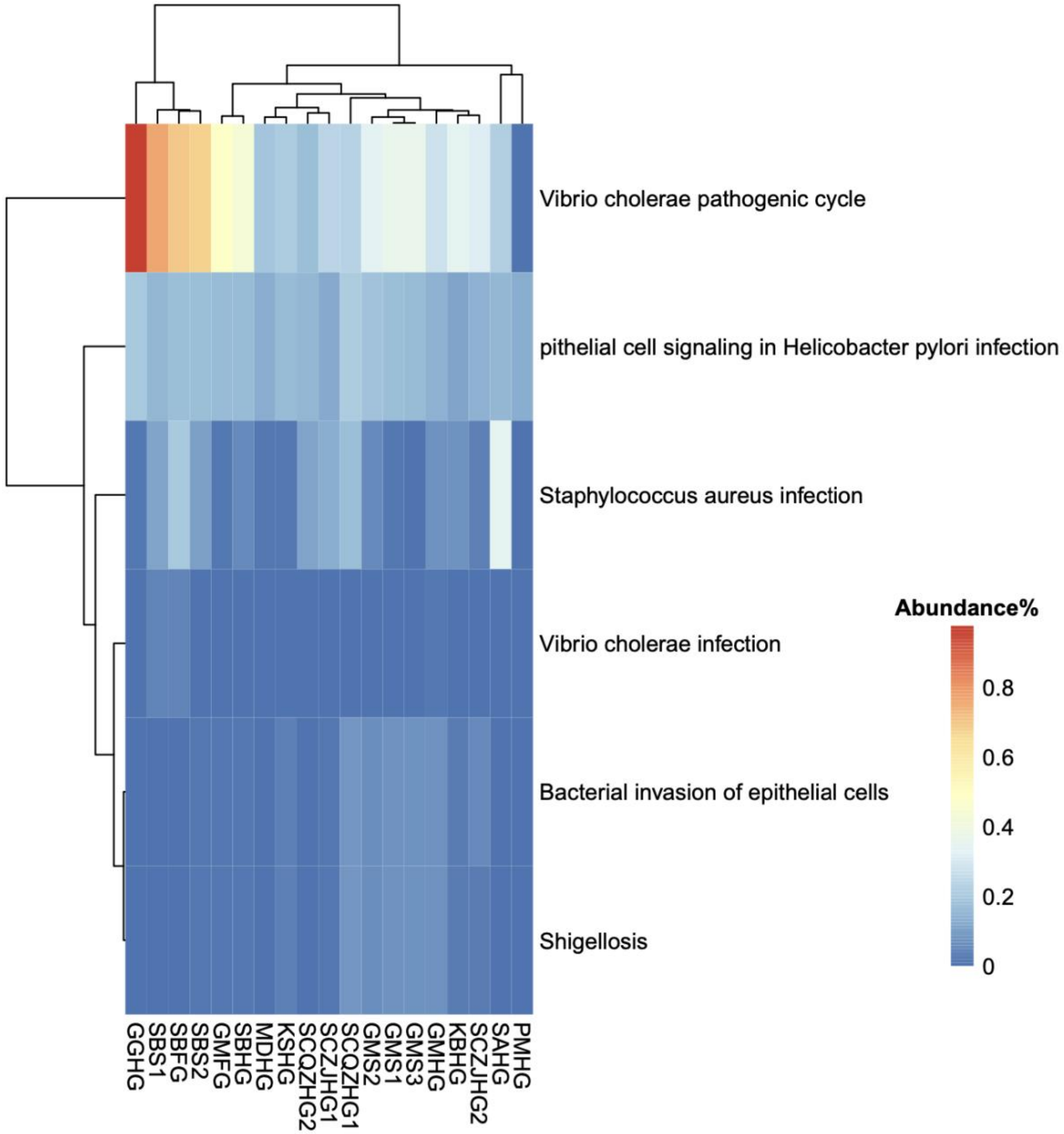

Figure S3. Relative abundance of the KEGG pathways related to pathogenic infection.

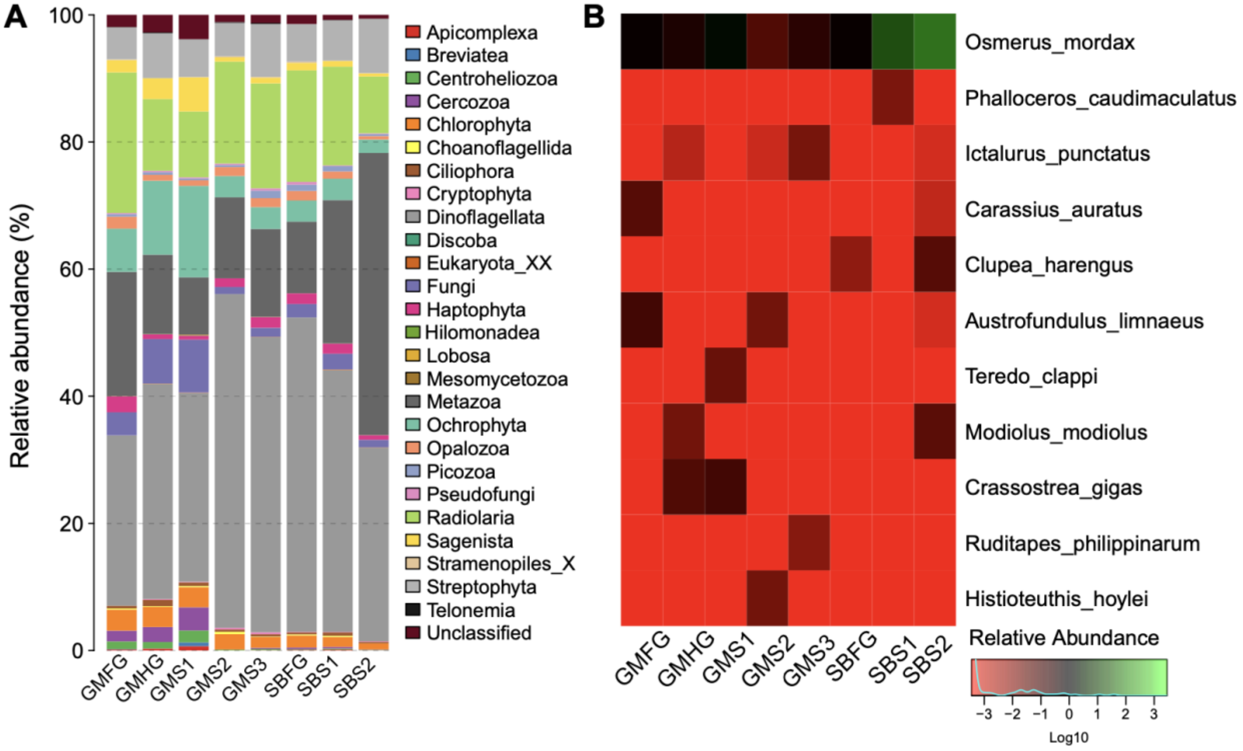

Figure S4. Eukaryotic community composition of the GM and SB gastrointestinal tract samples.

(A) Eukaryotic relative abundance at phylum level by using 18S rDNA amplicon sequencing. We identified a total of 26 eukaryotic phyla and found Dinoflagellata was the most dominant species (average = 38.79%), followed by Metazoa, Radiolaria, Streptophyta, Ochrophyta and Fungi with an average relative abundance of 18.22%, 14.78%, 6.55%, 6.05% and 3.41% across samples, respectively. The relative abundance of Dinoflagellata was significantly higher in the stomach samples of GM than that in the gut samples, whereas the relative abundance of Ochrophyta and Fungi was significantly lower in the stomach samples of GM than that in the gut samples, but Dinoflagellata was significantly lower in the stomach samples of SB than that in the gut sample. (B) Heatmap of the relative abundances of the fish, mollush and cephalopod species for the gastrointestinal tract samples. We identified a variety of fish among all samples, including rainbow smelt (Osmerus mordax), dusky millions fish (*Phalloceros caudimaculatus*), channel catfish (*Ictalurus punctatus*), goldfish (*Carassius auratus*), Atlantic herring (*Clupea harengus*), and *Austrofundulus limnaeus*. And we also detected some mollush species in the two delphinids and the cephalopod species *Histioteuthis hoylei* in the short-finned pilot.

Supplementary Tables

Table S2. Sample sequencing details in this study.

#SampleID	Paired_Clean_reads	Connect_tags	Clean_tags	ZOTUs
MDHG	99,612	95,413	94,428	38
PMHG	118,125	111,463	93,793	242
SCQZHG1	109,153	95,162	92,907	73
SCQZHG2	105,183	10,882	99,700	104
SAHG	93,859	90,765	89,086	163
GGHG	101,083	98,509	96,459	176
KBHG	13,964	13,472	5,508	106
SCZJHG1	9,056	8,621	8,194	125
SCZJHG2	94,870	90,943	88,360	156
KSHG	95,732	90,332	86,567	200
SBHG	19,112	17,113	11,797	23
GMHG	5,119	4,742	4,509	97
GMFG	95,420	92,971	90,500	34
GMS1	95,361	92,505	89,592	63
GMS2	95,902	93,149	90,094	58
GMS3	96,207	93,329	90,652	49
SBFG	21,500	19,716	18,773	63
SBS1	25,121	24,209	23,578	83
SBS2	25,692	24,616	23,659	78

Table S3. The significantly different KEGG pathways detected by using edgeR.

Kegg Pathway	P- Value	Enriched group	MDHG	KBHG	KSHG	PMHG	GMHG	SCQZHG1	SCQZHG2	SCZJHG1	SCZJHG2	SAHG	GGHG	SBHG
Other glycan degradation	5.85E-05	physeteroids and ziphiid	5.78	0.15	0.82	1.72	0.43	0.58	0.20	0.61	0.23	0.19	0.39	0.38
Glycosaminoglycan degradation	2.62E-04	physeteroids and ziphiid	2.98	0.02	0.21	0.41	0.10	0.07	0.01	0.09	0.07	0.02	0.23	0.11
Phosphotransferase system (PTS)	3.16E-04	delphinids	0.19	0.55	0.66	0.11	0.75	1.46	1.38	1.40	0.94	1.12	1.06	0.74
Histidine metabolism	8.32E-04	physeteroids and ziphiid	1.26	1.10	1.29	1.43	0.88	1.15	1.00	1.09	0.97	0.86	1.09	0.89
Glycine, serine and threonine metabolism	3.67E-3	physeteroids and ziphiid	0.99	1.32	1.16	1.22	1.04	0.82	0.71	0.96	0.89	0.96	1.05	1.05
Glyoxylate and dicarboxylate metabolism	9.15E-3	physeteroids and ziphiid	0.66	1.01	0.65	0.64	0.62	0.50	0.32	0.52	0.48	0.40	0.67	0.58
Citrate cycle (TCA cycle)	9.95E-3	physeteroids and ziphiid	1.17	0.99	0.85	1.55	0.99	0.66	0.67	0.67	0.76	1.03	0.92	1.04
Sphingolipid metabolism	1.14E-02	physeteroids and ziphiid	1.01	0.10	0.25	0.40	0.14	0.20	0	0.28	0.05	0.03	0.15	0.07
D-Alanine metabolism	1.20E-02	delphinids	1.26	1.33	1.75	1.43	1.82	2.12	2.10	1.79	1.85	2.67	1.49	1.86
Glycerophospholipid metabolism	1.31E-02	delphinids	0.38	0.62	0.62	0.42	0.65	0.76	0.73	0.67	0.72	0.74	0.71	0.66

Table S4. Relative abundance of 24 zOTUS assigned to "animal_parasites_or_symbionts" and "human_pathogens_all".

#ZOTUID	MDHG	KBHG	KSHG	PMHG	GMHG	SCQZHG1	SCQZHG2	SCZJHG1	SCZJHG2	SAHG	GGHG	SBHG	Taxonomy	Group
ZOTU4	0	0.47	8.07	0	17.80	21.71	8.02E-3	3.86	4.76	0.46	1.03E-3	2.42	Escherichia	animal_parasites_or_symbionts<br>human_pathogens_all
ZOTU58	0	0	0.01	0	0.08	0	0	0	0	2.90	4.12E-3	0	Actinobacillus	animal_parasites_or_symbionts
ZOTU118	0	0	0	0	0	0	0.59	0.02	0	0	5.15E-3	0	Actinobacillus	animal_parasites_or_symbionts
ZOTU184	0	0.67	0	0	0.11	0	2.01E-3	3.60	0.02	0	6.18E-3	0	Prevotella	animal_parasites_or_symbionts
ZOTU255	0	0.59	1.14E-3	1.05E-3	0.38	0	6.02E-3	1.92	7.42E-3	2.24E-3	1.03E-3	0	Actinobacillus	animal_parasites_or_symbionts
ZOTU266	0	0.47	0	0	0.19	0	4.01E-3	1.77	0.01	1.12E-3	0	0	Actinobacillus	animal_parasites_or_symbionts
ZOTU259	0	0	0	1.05E-3	0.11	0	1.00E-3	2.09	0	1.12E-3	1.03E-3	0	Prevotella	animal_parasites_or_symbionts
ZOTU358	0	0.43	0	0	0.38	0	7.02E-3	0.74	1.06E-3	3.37E-3	1.03E-3	0	Pasteurellaceae	animal_parasites_or_symbionts
ZOTU506	0	0.06	0	0	0	0	1.00E-3	1.25	0	0	1.03E-3	0	Roseburia	animal_parasites_or_symbionts
ZOTU305	0	0.08	0	0	0.11	0	0	0.83	7.42E-3	0	2.06E-3	0	Prevotella	animal_parasites_or_symbionts
ZOTU399	0	0	0	0	0	0	0	1.01	1.06E-3	1.12E-3	0	0	Prevotella	animal_parasites_or_symbionts
ZOTU419	0	0.53	0	0	0.08	0	2.01E-3	0.41	2.12E-3	2.24E-3	1.03E-3	0	Campylobacter	animal_parasites_or_symbionts
ZOTU481	0	0.14	0	0	0	0	0	0.60	1.06E-3	0	4.12E-3	0	Prevotella	animal_parasites_or_symbionts
ZOTU400	0	0	0	0	0.08	0	0	0.72	0	0	0	0	Prevotella	animal_parasites_or_symbionts
ZOTU407	0	0.08	0	0	0	0	0	0.66	0	0	0	0	Prevotella	animal_parasites_or_symbionts
ZOTU490	0	0.22	0	0	0.11	0	0	0.39	3.18E-3	0	1.03E-3	0	Pasteurellaceae	animal_parasites_or_symbionts
ZOTU444	0	0.06	0	0	0	0	0	0.41	0	0	1.03E-3	0	Prevotella	animal_parasites_or_symbionts
ZOTU555	0	0	0	0	0	0	0	0.44	0	0	0	0	Prevotella	animal_parasites_or_symbionts
ZOTU616	0	0	0	0	0	0	0	0.39	0	0	1.03E-3	0	Prevotella	animal_parasites_or_symbionts

ZOTU497	0	0.14	0	0	0.08	0	2.01E-3	0.09	7.42E-3	0	0	0	Pasteurellaceae	animal_parasites_or_symbionts
ZOTU552	0	0.10	0	0	0	0	0	0.30	0	0	0	0	Prevotella	animal_parasites_or_symbionts
ZOTU613	0	0.14	0	0	0	0	1.00E-3	0	0.01	1.12E-3	0	0	Stenotrophomonas	animal_parasites_or_symbionts human_pathogens_all
ZOTU619	0	0	0	0	0	0	0	0.32	0	0	0	0	Prevotella	animal_parasites_or_symbionts
ZOTU617	0	0	0	0	0	0	0	0.24	0	0	0	0	Prevotella	animal_parasites_or_symbionts

Table S5. Summary of operational taxonomic unit (OTU) annotations for the 12S rDNA amplicon of two dolphin species.

#OTU_ID	GMFG	GMIF	GMS1	GMS2	GMS3	SBS1	SBS2	SBFG	Taxonomy
Otu0034	795	0	0	0	0	0	0	0	Metazoa; Chordata; Actinopteri; Perciformes; Cottidae; Trachidermus; Trachidermus_fasciatus
Otu0046	0	0	0	0	0	118	134	0	Metazoa; Chordata; Actinopteri; Clupeiformes; Clupeidae; Clupea; Clupea_pallasii
Otu0053	0	0	0	0	0	30	171	0	Metazoa; Chordata; Actinopteri; Osmeriformes; Salangidae; Mallotus; Mallotus_villosus
Otu0092	0	0	0	0	0	0	0	10	Metazoa; Chordata; Actinopteri; Perciformes; Sebastidae; Sebastiscus; Sebastiscus_marmoratus
Otu0095	10	5	0	1	7	10	7	6	Metazoa; Chordata; Actinopteri; Siluriformes; Auchenipteridae; Tatia; Tatia_intermedia
Otu0101	0	0	0	8	0	0	0	10	Metazoa; Chordata; Actinopteri; Tetraodontiformes; Tetraodontidae; Takifugu; Takifugu_obscurus
Otu0113	0	0	6	0	0	0	0	0	Metazoa; Chordata; Actinopteri; Cypriniformes; Danionidae; Danio; Danio_rerio
